# Pharmacological Stimulation of Nurr1 Promotes Cell Cycle Progression in Adult Hippocampal Neural Stem Cells

**DOI:** 10.3390/ijms21010004

**Published:** 2019-12-18

**Authors:** Haena Moon, Seong Gak Jeon, Jin-il Kim, Hyeon soo Kim, Sangho Lee, Dongok Kim, Seungjoon Park, Minho Moon, Hyunju Chung

**Affiliations:** 1 Department of Core Research Laboratory, Medical Science Research Institute, Kyung Hee University Hospital at Gangdong, Seoul 134-727, Korea; godgosk@gmail.com (H.M.); lshkidney@khu.ac.kr (S.L.); kdw@khnmc.or.kr (D.K.); 2 Department of Biochemistry, College of Medicine, Konyang University, Daejeon 35365, Korea; jsg7394@naver.com (S.G.J.); sooya1105@naver.com (H.s.K.); 3 Department of Nursing, College of Nursing, Jeju National University, Jeju 63243, Korea; neoreva@hanmail.net; 4 Department of Biomedical Science, Graduate School, Kyung Hee University, Seoul 02447, Korea; sjpark@khu.ac.kr

**Keywords:** Nurr1, amodiaquine, cell cycle, neurogenesis, hippocampus

## Abstract

Nuclear receptor related-1 (Nurr1) protein performs a crucial role in hippocampal neural stem cell (hNSC) development as well as cognitive functions. We previously demonstrated that the pharmacological stimulation of Nurr1 by amodiaquine (AQ) promotes spatial memory by enhancing adult hippocampal neurogenesis. However, the role of Nurr1 in the cell cycle regulation of the adult hippocampus has not been investigated. This study aimed to examine changes in the cell cycle-related molecules involved in adult hippocampal neurogenesis induced by Nurr1 pharmacological stimulation. Fluorescence-activated cell sorting (FACS) analysis showed that AQ improved the progression of cell cycle from G_0_/G_1_ to S phase in a dose-dependent manner, and MEK1 or PI3K inhibitors attenuated this progression. In addition, AQ treatment increased the expression of cell proliferation markers MCM5 and PCNA, and transcription factor E2F1. Furthermore, pharmacological stimulation of Nurr1 by AQ increased the expression levels of positive cell cycle regulators such as cyclin A and cyclin-dependent kinases (CDK) 2. In contrast, levels of CDK inhibitors p27^KIP1^ and p57^KIP2^ were reduced upon treatment with AQ. Similar to the in vitro results, RT-qPCR analysis of AQ-administered mice brains revealed an increase in the levels of markers of cell cycle progression, PCNA, MCM5, and Cdc25a. Finally, AQ administration resulted in decreased p27^KIP1^ and increased CDK2 levels in the dentate gyrus of the mouse hippocampus, as quantified immunohistochemically. Our results demonstrate that the pharmacological stimulation of Nurr1 in adult hNSCs by AQ promotes the cell cycle by modulating cell cycle-related molecules.

## 1. Introduction

The nuclear receptor-related 1 protein (Nurr1, *NR4A2*), a member of nuclear receptor subfamily 4A (NR4A), is expressed abundantly throughout the central nervous system during the developmental stage and adulthood [1,2,3,4,5]. Nurr1 is known to perform pivotal roles in the development and protection of dopaminergic neurons [5,6,7,8]. In addition, Nurr1 is involved in a variety of cognitive functions such as learning and memory [9,10,11,12,13,14]. In particular, Nurr1 haploinsufficiency is associated with cognitive and language impairment in humans [15,16]. Furthermore, the intracellular transcription factor Nurr1 has been suggested as a therapeutic target since it undergoes alterations and is involved in the pathology of neurodegenerative diseases including Parkinson’s disease (PD) and Alzheimer’s disease (AD) [17,18,19,20,21,22,23]. Despite the fact that Nurr1 is considered an orphan nuclear receptor, the endogenous ligands for Nurr1 have not yet been identified [1,2,24]. However, amodiaquine (AQ) is reported as a pharmacological agonist showing direct interactions with the Nurr1 ligand-binding domain and upregulation of Nurr1 transcriptional activity [25]. Moreover, in rodent models of PD and AD, AQ administration alleviates disease-related pathogenesis through Nurr1 activation [18,25]. Remarkably, AQ not only promotes midbrain dopaminergic neurogenesis, but also restores impaired hippocampal neurogenesis in AD animal models [18,25].

The adult brain neurogenesis relies on the coordination of cellular processes, including proliferation, cell cycle, survival/death pathway, migration, differentiation, and maturation [26]. Among these, the expression of cell cycle-related molecules has been investigated extensively in embryonic development as it offers important clues for the neurogenic process [27,28,29,30]. Although some cell cycle/neurogenesis mechanisms are common to both the developmental stage and adult brain, fundamental differences in environmental maturation and functional activity exist in the neurogenic niche, the hippocampus [28,30]. Hippocampal neurogenesis occurs during adulthood in the dentate gyrus (DG) subgranular zone (SGZ) in the hippocampus, a critical brain region associated with the regulation of cognitive functions such as memory and learning [31,32,33]. These hippocampal adult-born neurons, generated through proliferation, differentiation, and maturation, contribute to learning, spatial, and cognitive memory through functional integration [34,35,36,37]. Previously, we demonstrated that the treatment of adult rats and SGZ of adult mice with the Nurr1 agonist AQ stimulates the proliferation and neuronal differentiation in both the hippocampal neural stem cells (hNSCs) [38]. In particular, AQ-induced neurogenesis leads to an improvement in both short- and long-term memory. The pharmacological stimulation of Nurr1 by AQ induces the phosphorylation of Akt and extracellular signal-regulated protein kinases 1 and 2 (Erk1/2) [38]. Although an understanding of neurogenesis mechanisms could provide insight into cell-based therapeutic strategies for neurodegenerative diseases [39,40,41], the precise mechanisms underlying the neurogenic effects of Nurr1 remain unclear.

Several cell cycle-related molecules and signaling pathways are involved in the regulation of hippocampal neurogenesis [26]. The cell cycle consists of four consecutive phases as follows. The first gap (G_1_) phase to prepare for DNA synthesis, the synthetic (S) phase for DNA replication, the second gap (G_2_) to prepare for mitosis, and the mitosis (M) phase [42]. Cyclins, as major regulators of the cell cycle and the activity of cyclin-dependent kinases (CDK), coordinate the checkpoints of the cell cycle [43]. During the G1 phase, CDK4/6 complexes with cyclin D for activation, and increases the NSCs pool [44,45]. CDK2 complexes with cyclin A and E as a catalytic enzyme, which is necessary for the G_1_ phase progression and G_1_ to S phase. The activity of these cyclin-CDK complexes can be suppressed by interaction with the CDK inhibitory proteins, such as the INK4 family and the CIP/KIP family. Unlike the INK4 family (p16^INK4a^, p15^INK4b^, p18^INK4c^, and p19^INK4d^), which inhibits the catalytic subunit CDK4/6 primarily, the CIP/KIP family (p21^Cip1^, p27^KIP1^, and p57 ^KIP2^) inhibits cell cycle progression by attaching to both cyclins and CDK subunits [45,46,47]. Most neuronal cells in the adult mammalian brain are in a quiescent differentiated G_0_ phase, probably involving region-specific CDK inhibitors [48,49]. In addition, several studies have shown the involvement of cell cycle-related molecules in neuronal migration, maturation, plasticity, and dendrite development [50,51]. Therefore, the balance between the cyclin-CDK complexes and CDK inhibitors performs a pivotal role in cell cycle progression and adult neurogenesis [26].

Although pharmacological stimulation of Nurr1 has been shown to augment hippocampal neurogenesis in normal and neurodegenerative (AD and PD) models, the effect of Nurr1 on cell cycle regulators in adult hippocampal neurogenesis is not understood fully. Hence, we hypothesize that the Nurr1 proliferative effect on adult hippocampal neurogenesis may be related to the modulation of cell cycle-related molecules. In this study, we showed that Nurr1 pharmacological stimulation by AQ promoted cell cycle progression in both hippocampal NSCs and the mouse hippocampus.

## 2. Results

### 2.1. AQ Stimulates the Proliferation and Cell Cycle Progression of Adult Rat hNSCs

**Figure 1:**
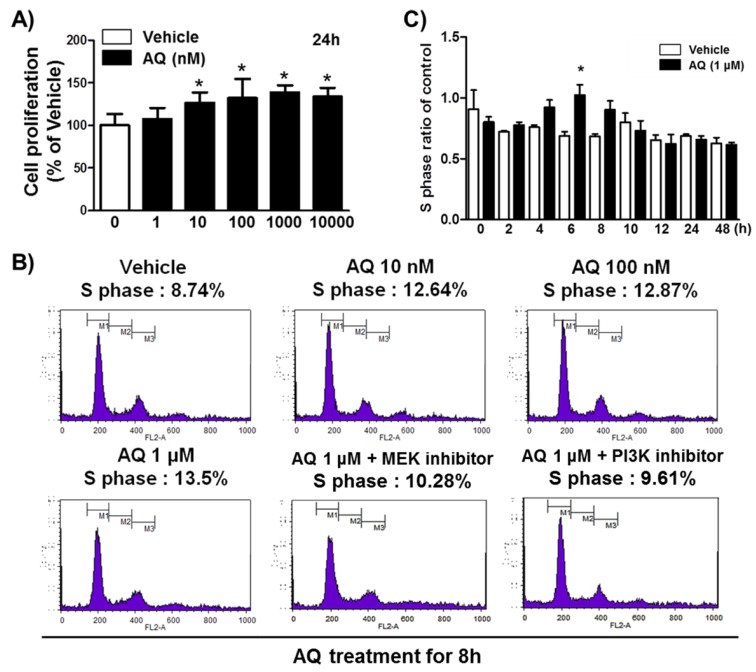
Amodiaquine (AQ) stimulates adult rat hNSC cell cycle progression. (**A**) Cells were treated with 1, 10, 100, 1000, and 10,000 nM AQ for 24 h, and cell proliferation was evaluated by MTT assay. (**B**) Cells were treated with 10 nM, 100 nM, and 1 μM AQ for 8 h with or without MEK and PI3K inhibitor, and the percentage of S phase cells was analyzed by FACS analysis. (**C**) After cell treatment with 1 μM AQ, the S phase ratio of cells was analyzed time-dependently over 48 h by FACS and compared with the vehicle-treated control (* *p* < 0.05 compared with vehicle-treated control, three independent cell culture preparations).

In our previous study, we demonstrated that Nurr1 pharmacological stimulation by AQ promoted adult neurogenesis in both the mouse hippocampus and adult rat hNSCs [38]. Therefore, we performed an MTT assay to confirm adult rat hNSC proliferation in the presence of varying AQ concentrations. Consistent with our previous results, in this study, 24 h AQ treatment increased cell proliferation significantly in a dose-dependent manner from 10 to 1000 nM AQ (Figure 1A). FACS analysis was conducted to evaluate the effect of AQ treatment on the S phase distribution of cells in the cell cycle. Adult rat hNSCs were incubated for 16 h without growth factors and treated for 8 h with vehicle or AQ (10 nM, 100 nM, and 1 μM). In total, 8.74% of the vehicle-treated cells were distributed in the S phase, whereas the S phase population in the AQ-treated cells dose-dependently increased to 13.5% at 1 μM (Figure 1B). As our previous findings showed that the proliferation effect of AQ is mediated by the Erk1/2 and Akt signaling pathways [38], we investigated the effect of MEK and PI3K inhibitors on the increased S phase population after AQ treatment. As expected, the AQ treatment resulted in increased S phase cell cycle distribution, and treatment with either MEK (10.28%) or PI3K (9.61%) inhibitors attenuated this effect (Figure 1B). An analysis of the ratio of the S phase cell cycle progression at 48 h in AQ-treated cells showed a significantly higher S phase distribution than that in vehicle-treated cells at 6 h (Figure 1C). However, AQ treatment did not alter cell cycle ratios in other cell cycle phases markedly (Figure S1). These results suggest that AQ treatment of adult rat hNSCs not only stimulates cell proliferation, but also promotes cell cycle progression through the MEK and PI3K signaling pathways.

### 2.2. AQ Upregulates the Levels of Cell Cycle-Related Markers MCM5 and PCNA

**Figure 2:**
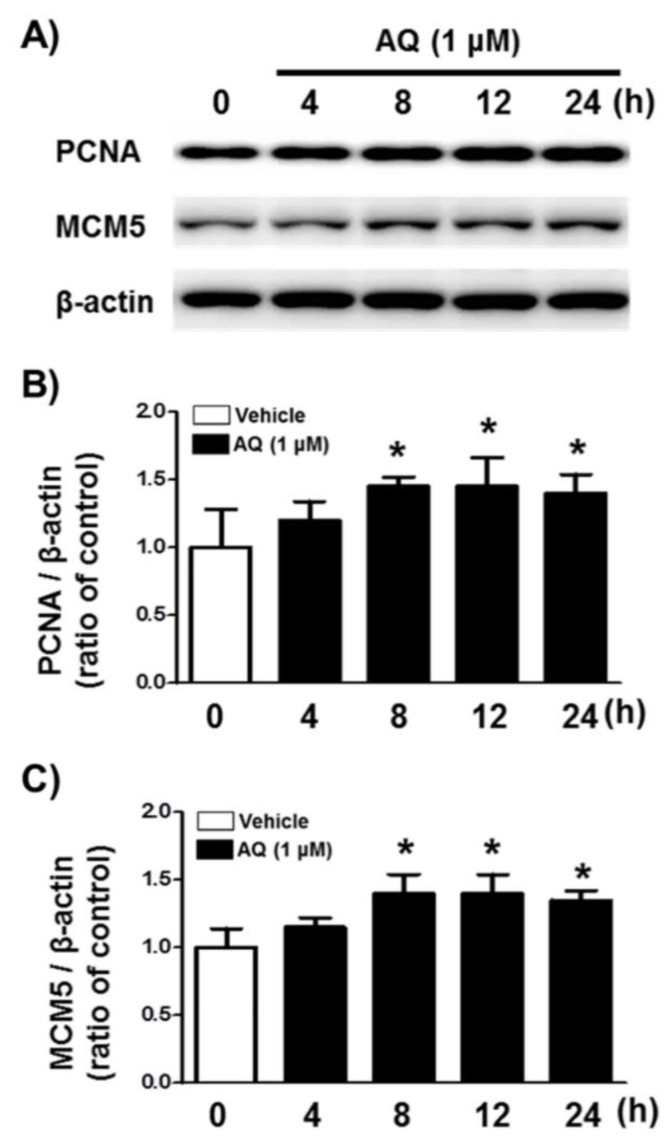
Amodiaquine (AQ) increases the expression of MCM5 and PCNA in adult rat hNSCs. (**A**) Cells were treated with 1 μM AQ for 4, 8, 12, and 24 h. Cell lysates were examined by western blotting using anti-PCNA, MCM5, and β-actin antibodies. Quantified PCNA (**B**) and MCM5 (**C**) band intensities were normalized to β-actin band intensity. The bar graphs show band intensity as a ratio of the vehicle-treated control (* *p* < 0.05 compared with vehicle-treated control, three independent cell culture preparations).

We analyzed PCNA and MCM5 levels, well-established markers of DNA replication, and cell cycle progression [52,53,54,55] by western blotting to demonstrate AQ role in stimulating proliferation and cell cycle progression (Figure 2A). After 8 h of AQ (1 μM) treatment, both PCNA and MCM5 protein levels increased significantly over 24 h (Figure 2B,C). These results indicate that AQ-stimulated cell cycle progression is accompanied by the upregulation of MCM5 and PCNA, which are essential for mitotic progression.

### 2.3. AQ Enhances the Nuclear Expression of E2F1 in a Nurr1-Dependent Manner

**Figure 3:**
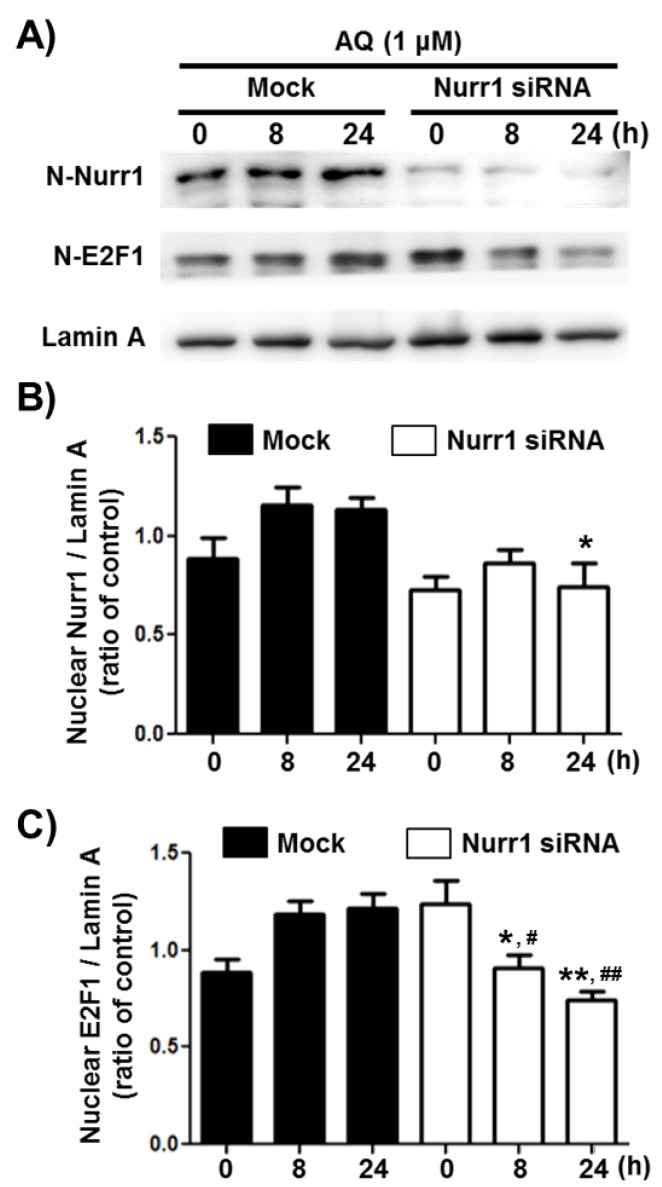
Amodiaquine (AQ) increases the nuclear expression of the E2F1 transcription factor via Nurr1 in adult rat hNSCs. (**A**) Nurr1 siRNA or Mock transfected cells were treated with 1 μM AQ for 8 and 24 h with or without Nurr1 siRNA transfection. The nuclear fractions of cell lysates were analyzed by western blotting using anti-E2F1, Nurr1, and lamin A antibodies. Quantified Nurr1 (**B**) and E2F1 (**C**) band intensities were normalized to lamin A band intensity. The bar graphs represent the mean intensity of the protein bands displayed as fold change of Nurr1 or E2F1 / Lamin A ratio (* *p* < 0.05, ** *p* < 0.01 compared with mock group for each time point, # *p* < 0.05, ## *p* < 0.01 compared with mock group at 0 h).

Transcription factor E2F1 is a significant regulator of neurogenesis and cell cycle progression via induction of genetic expressions associated with proliferation and differentiation [49,56,57,58]. To investigate if Nurr1 mediates AQ-induced cell cycle progression, the E2F1 protein levels in the nuclear fraction of adult rat hNSCs, after AQ treatment and Nurr1 siRNA transfection, were analyzed by western blotting (Figure 3A). The enhanced nuclear expression of Nurr1 by AQ treatment (1 μM) was silenced considerably after transfection with Nurr1 siRNA (Figure 3B). The nuclear expression of E2F1 increased time-dependently after treatment with AQ (1 μM). In contrast, Nurr1 siRNA-transfected adult rat hNSCs suppressed the AQ treatment-induced E2F1 increase (Figure 3C). These results demonstrate that Nurr1 mediates the increased expression of E2F1 after AQ treatment.

### 2.4. AQ Promotes Cell Cycle Progression by Regulating Cell Cycle-Related Molecules

**Figure 4:**
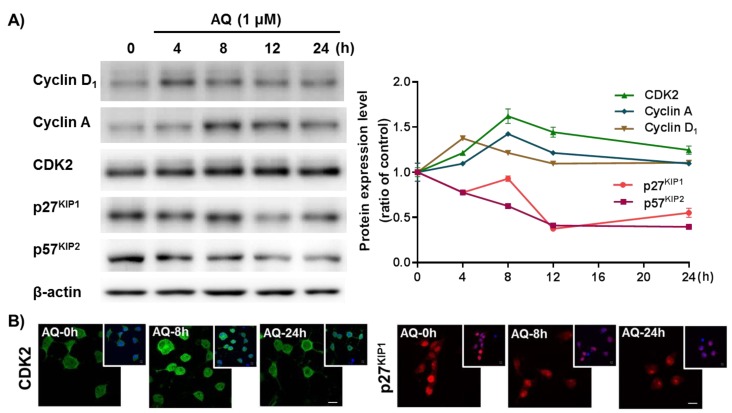
Amodiaquine (AQ) modulates the expression of cell cycle-related molecules in adult rat hNSCs. (**A**) Cells were treated with 1 μM AQ for 4, 8, 12, and 24 h. Cell lysates were analyzed by western blotting using anti-Cyclin D_1_, cyclin A, CDK2, p27^KIP1^, p57^KIP2^, and β-actin antibodies. (**B**) Cells were treated with 1 μM AQ for 8 and 24 h, and the CDK2- and p27^KIP1^-positive cells were visualized by confocal microscopy for immunocytochemical analysis. The small panel on the top right is a merged image of DAPI counterstained nuclei. Scale bar: 10 μm.

The cell cycle mechanism of AQ-mediated proliferation was evaluated in adult rat hNSCs after AQ treatment by time-dependent changes in cell cycle-related molecules. Cyclin D_1_ releases the E2F1 transcription factor by phosphorylating the retinoblastoma (Rb) protein to regulate cell cycle progression [59,60,61]. In addition, cyclin A accumulation during the S phase is mediated by the E2F1 transcription factor [62,63]. Furthermore, CDK2 is not only essential for cyclin D_1_-expressing cell survival, but also forms a cyclin A/CDK2 complex, a crucial factor necessary for cell proliferation and division [64,65,66]. These cell cycle positive modulators (cyclin D_1_, cyclin A, and CDK2) were analyzed after time-dependent AQ treatment by Western blotting. Cyclin D_1_ protein levels increased 4 h after AQ treatment. Sequentially, an increase in cyclin A and CDK2 levels were observed 8 h after AQ treatment (Figure 4A). The CDK inhibitors p27^KIP1^ and p57^KIP1^ are important negative regulators of the cell cycle for inducing cell cycle exit [67,68,69]. Time-dependent analyses of p27^KIP1^ and p57^KIP1^ protein levels in AQ-treated cells using Western blotting showed a time-dependent reduction in both proteins (Figure 4A). In addition, the progressive role of AQ in the cell cycle was confirmed by immunocytochemistry analysis of CDK2 and p27^KIP1^ using AQ-treated adult rat hNSCs. As shown in Figure 4A, AQ enhanced CDK2 expression and decreased the nuclear expression of p27^KIP1^ in adult rat hNSCs (Figure 4B). These results demonstrate that AQ treatment can promote cell cycle progression through the regulation of cell cycle-related molecules in adult rat hNSCs.

### 2.5. AQ Administration Upregulates Cell Cycle-Dependent Gene Expression in Mouse Brains

**Figure 5:**
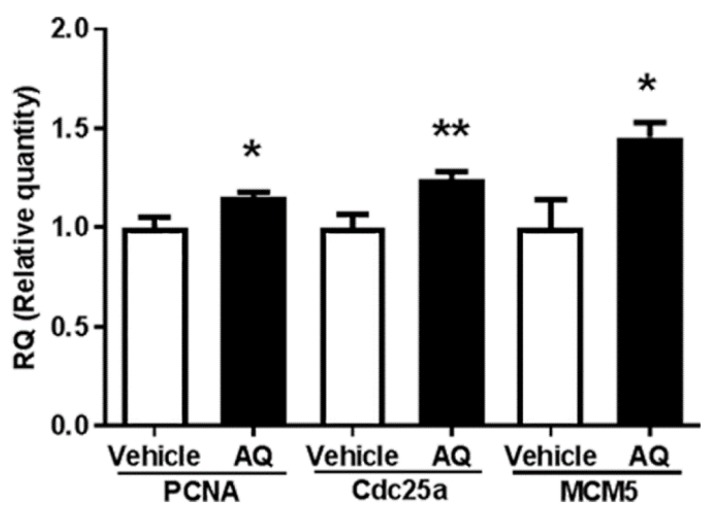
Amodiaquine (AQ) increases the expression of cell cycle-dependent gene expression in adult mouse brains. C57BL/6 mice were euthanized after two weeks of AQ administration, and RNA was extracted from the whole brain lysates for RT-qPCR analysis. RT-qPCR was performed to quantitate mRNA levels of PNCA, Cdc25a, and MCM5 and normalized to β-actin. The bar graphs represent the relative quantity standardized with the saline-administered group (* *p* < 0.05 and ** *p* < 0.01 compared with the vehicle-treated group, six mice per group).

The in vivo effect of AQ on cell cycle progression was investigated by evaluating the expression of the cell cycle-dependent genes PCNA, Cdc25a, and MCM5 in the brains of AQ-administered mice by RT-qPCR. Similar to PCNA and MCM5, Cdc25A was shown to be a critical regulator of G_1_ to S phase transition during cell cycle progression [70,71]. The expression of PCNA, Cdc25a, and MCM5 in the AQ-administered mice increased significantly compared to that in the vehicle-administered mice (Figure 5). These results are consistent with the above in vitro results.

### 2.6. AQ Promotes Cell Cycle Progression in the Dentate Gyrus of Mouse Hippocampi

**Figure 6:**
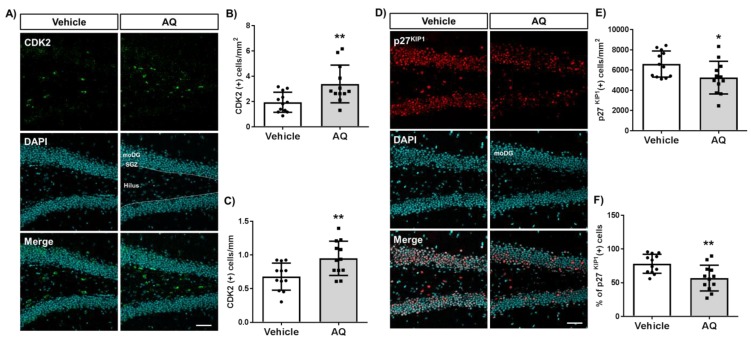
Amodiaquine (AQ) modulates the expression of CDK2 and p27KIP1 in the dentate gyrus (DG) of the hippocampus. (**A**) CDK2-positive cells in the DG of the hippocampus of AQ-administered mouse brains were visualized by immunohistochemistry. (**B**) The number of CDK2 (+) cells per area of hilus increased significantly in the DG of AQ-administered mice. (**C**) The number of CDK2 (+) cells per length of the subgranular zone (SGZ) increased substantially in the DG of AQ-administered mice. **(D**) The p27KIP1-positive cells in the DG of the hippocampus were visualized by immunohistochemistry of the AQ-administered mouse brains. (**E**) The number of p27^KIP1^ (+) cells per area of the molecular layer (moDG) decreased significantly in the DG of AQ-administered mice. (**F**) The percentage of p27KIP1 (+) cells in the total cells was markedly reduced in the DG of AQ-administered mice (* *p* < 0.05 and ** *p* < 0.01 compared with the vehicle-treated group, six mice per group). Scale bar: 50 μm.

We investigated whether the observed in vitro changes in cell cycle molecules were also present in the AQ-administered mice hippocampi by immunohistochemical staining of the cell cycle positive regulator CDK2 (Figure 6A) and negative regulator p27^KIP1^ (Figure 6D). The number of CDK2-positive cells per hilus area in the DG increased significantly in AQ-administered mice (Figure 6B). In addition, the number of CDK2-positive cells per length of SGZ increased substantially after the AQ administration (Figure 6C). In contrast, the number of p27^KIP1^-positive cells per molecular layer area of DG (moDG) in the hippocampus decreased significantly in AQ-administered mice (Figure 6E). Furthermore, the percentage of p27^KIP1^-positive cells from the DAPI-stained cells in DG decreased significantly after AQ administration (Figure 6F). These results demonstrate, for the first time, that the administration of AQ promotes cell cycle by positively modulating cell cycle-related molecules, such as CDK2 and p27^KIP1^, in the DG of the hippocampus.

## 3. Discussion

This study aimed to investigate the pharmacological effect of AQ-induced Nurr1 stimulation on hNSC cell cycle progression. Our results showed that Nurr1 pharmacological stimulation by AQ increased hNSC proliferation significantly, coherent with data of earlier studies [18,25,38]. Furthermore, this study showed that pharmacological stimulation of Nurr1, positively regulated cell cycle-related molecules and markers of cell cycle progression in adult hNSCs, as demonstrated by histological quantification and visualization in the adult mouse brains.

An increase in the S phase ratio after AQ treatment was observed for the first time. In addition, this effect was diminished by inhibitors of PI3K and MEK, upstream signaling molecules of Akt, and Erk1/2 in adult hNSCs (Figure 1). These results suggest that AQ promotes cell cycle progression through MEK and PI3K pathways. Phosphorylated Akt and Erk1/2 are important signaling molecules in adult hippocampal neurogenesis [38,72,73,74,75]. Considering that the increased S phase entry in neural progenitor cells of the subventricular zone is associated with neurometabolic-vascular coupling [76] the enhanced AQ effect on the S phase ratio of hNSCs may promote adult hippocampal neurogenesis, as shown in a previous study [38]. Therefore, the study findings support earlier results, that AQ stimulates the Erk1/2 and Akt signaling pathway [38], and further shows that Nurr1 mediates the pharmacological action of AQ by modulating the S phase of the cell cycle.

The markers influencing the AQ-mediated cell cycle changes were examined after AQ treatment by monitoring changes in the expression level of cell proliferation marker molecules MCM5 and PCNA. The expression of both MCM5 and PCNA increased after 8 h of AQ treatment (Figure 2 and Figure S3). Consistently, in vivo analysis of gene expression changes in cell cycle-specific proteins also showed that AQ administration promoted gene expression of molecules participating in G_1_/S transitions, such as PCNA, Cdc25a, and MCM5 (Figure 5). MCM5 is a well-established cell proliferation marker and a component of the MCM complex, which functions in DNA replication and RNA transcription [77,78,79]. Since the MCM family activates the G_0_ to G_1_/S transition in the cell cycle, the upregulated expression of MCM5 might lead to increased S phase levels after AQ treatment. In addition, PCNA, another universal cell proliferation marker, is associated with replication of DNA as well as regulation of cell cycle and is an essential factor in G_1_ progression and transition of G_1_/S phase transition [80]. Collectively, the results on increased levels of markers participating in S phase stimulation corroborate the increase in the S phase ratio by AQ treatment.

Consistent with the above results, AQ upregulated both Nurr1 and E2F1 expression, and the siRNA silencing of Nurr1 abolished this effect (Figure 3). E2F1 transcription factor interacts directly with the cyclin A-CDK2 complex to regulate the cell cycle and is significant for the G_1_ to S phase transition of cell cycle progression [42,81,82]. Moreover, MCM5 transcription, negatively regulated by p53, is controlled by E2F1 [53]. Therefore, increased MCM5 expression levels after AQ treatment could result from the upregulation of E2F1, which may be dependent on Nurr1 expression.

The time-dependent AQ treatment effects on cell cycle-related molecules were examined further in hNSCs. The cyclin D_1_ expression increased after four hours of 1 μM AQ treatment, and expression of cyclin A and CDK2 increased after eight hours of treatment (Figure 4). The critical roles of the Cyclin family and CDK in the regulation of the cell cycle are well established [83]. The cyclin D-Cdk4/6 complex phosphorylates Rb and is essential for the transition of G_1_/S phase [42,84,85]. In addition, cyclin D_1_ performs CDK-independent functions, including participation in transcription and differentiation, by acting on nuclear receptors such as PPARγ [86]. Moreover, cyclin D_1_ phosphorylates Rb, releasing E2F1 transcriptional factors [87,88]. Interestingly, among the cyclins, while cyclin D_2_ plays a significant role in spontaneous adult hippocampal neurogenesis [89], our results show that AQ-stimulated cell cycle progression is followed by the upregulation of cyclin D_1_ in the adult hippocampus (Figure 4A). These results indicate that AQ-induced cell cycle progression may involve additional pathways, other than the spontaneous adult hippocampal neurogenesis pathway. CDK2 is another critical factor functioning in cell cycle regulation. The cyclin E-CDK2 complex promotes the formation of a pre-replication complex and plays a critical role in G_1_ to S transition [90,91,92]. Cyclin A, another cyclin that binds to CDK2, forms the cyclin A-CDK2 complex and participates in S to G_2_ phase transition [93]. In addition, cyclin A controls DNA replication during the cell cycle [62]. The cyclin E-CDK2 complex also actively controls cell cycle progression via modulation of the CDK inhibitor p27^KIP1^ [94,95]. Further, p27^KIP1^ blocks G_1_ to S phase transition in a CDK-dependent and -independent manner [96]. Another CDK inhibitor, p57^KIP2^, functions as a negative modulator of the cell cycle by adjusting the transition of G_1_/S phase [97]. In addition, p57 blocks DNA replication via an interaction with and interference of the activity of PCNA [98]. In this study, p27^KIP1^ and p57^KIP2^, negative modulators of the cell cycle, decreased significantly after AQ treatment (Figure 4). These results suggest that the AQ-mediated Nurr1 activation could contribute to cell cycle progression by inhibiting the expression of negative modulators in the cell cycle.

**Figure 7:**
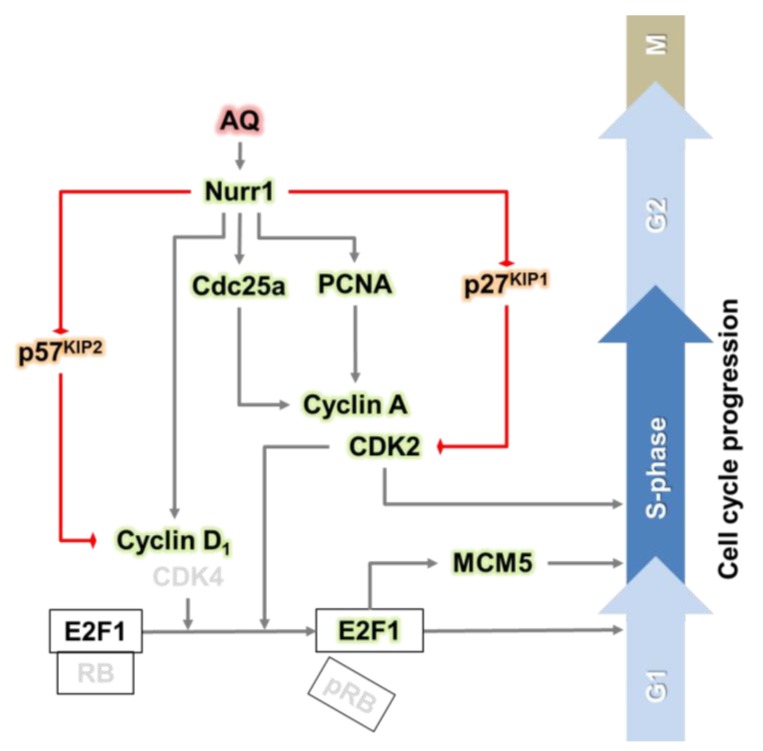
Graphical abstract: the pharmacological stimulation of Nurr1 promotes adult hippocampal neurogenesis.

Furthermore, the positive modulators for the cell cycle increased, while the negative modulators such as p27^KIP1^ and p57^KIP2^ significantly decreased after AQ mediated Nurr1 activation. Notably, the in vivo results on positive regulation of cell cycle progression by AQ in the DG of the hippocampus are consistent with the in vitro results, suggesting that AQ stimulates the S phase by regulating upstream molecules of E2F1 (Figure 5 and Figure 6). Therefore, the combined results suggest the involvement of a cell cycle-related molecular mechanism for the upregulation of the S phase by AQ in Nurr1-stimulated adult hNSCs (Figure 7). Nonetheless, our in vitro and in vivo results regarding the effect of AQ on neurogenesis should be interpreted with caution. Since there was difference in species between in vitro and in vivo studies, this discrepancy may cause a limitation for our conclusion. Thus, further studies using the same species might be noteworthy for elucidating more precise mechanisms of the cell cycle modulating effect of AQ.

Many studies have presented evidence for cell cycle dysregulation as a fundamental cause of neurodegenerative disease [99]. Some studies have suggested that cell cycle deficits, including abnormal cell cycle entry, may contribute to neurodegeneration [100,101,102]. Despite concerns regarding neuronal cell death due to abnormal cell cycle re-entry in mature neurons, the dynamic modulation of the cell cycle in the adult brain has been suggested as a curative point for neurodegenerative diseases [103,104]. Neuronal cell death results in reduced neural connectivity resulting in functional degeneration [105]. In addition, depletion of neurogenesis aggravates cognitive functions in AD [106]. Therefore, the cell cycle regulation of adult NSCs in the DG of the hippocampus is vital for the prevention and blocking the progression of neurodegenerative disease. Promoting adult neurogenesis, supplementing neurons, or increasing the number of neuronal cells may offer some therapeutic strategies for neurodegenerative disease therapy.

Attempts to treat neurodegenerative diseases, including AD and PD, were made using several pharmacological mechanisms [107]. Neurodegeneration outcomes, including neuronal cell death and synaptic loss, lead to a variety of neurological impairments, including cognitive dysfunction [108]. Therefore, triggering adult neurogenesis through the regulation of the cell cycle may be necessary for repairing neuronal loss due to neurodegenerative disease and improving functional impairments. In addition, the progressive reduction in adult neurogenesis with aging is well established in rodents [109,110,111].

A reduction in Nurr1 levels has been reported in several neurodegenerative diseases [18]. The importance of Nurr1 in PD pathology is well established [19], and reduced Nurr1 levels have been reported in AD animal models and the brains of patients with AD [112,113]. Therefore, Nurr1 has been suggested as a valid biomarker for neurodegenerative diseases and a therapeutic target [18,23]. In our studies, we reported that the pharmacological stimulation of Nurr1 could improve or restore cognitive function through adult hippocampal neurogenesis [23,38]. In this study, a new mechanism underlying Nurr1-mediated adult neurogenesis, i.e., through the regulation of cell cycle-related molecules, is suggested. Our study presents the possibility of pharmacological activation of Nurr1 for the treatment of neurodegenerative disease and provides new insights into the cell cycle-related neurogenic mechanism of Nurr1.

Previous studies indicated that the cell cycle-related role of Nurr1 in neurogenesis remains controversial. Nurr1 has been exhibited to induce cell cycle halt and contribute to neuronal differentiation and maturation, in particular, in developmental stages such as embryos and pups, and dopaminergic cell lines [114,115,116]. In addition, the expression of p57^KIP2^ required for the maturation of postmitotic differentiating dopamine cells was shown to be Nurr1-dependent [117]. Furthermore, the transfection of Nurr1 in embryonic olfactory bulb stem cells inhibits proliferation and increases tyrosine hydroxylase-positive immature neurons [118]. In contrast, this study demonstrated that the pharmacologic stimulation of Nurr1 by AQ in the hippocampus of mice and rat hNSCs induces proliferation and cell cycle progression. These results suggest the role of Nurr1 in cell cycle modulation depending on the cellular environment and also indicate the likelihood of cellular factors mediating or involving Nurr1-induced cell cycle regulation. Therefore, future studies will focus on investigating the changes in cell cycle-related molecules in Nurr1 expressing cell types and brain regions following various AQ treatment regimes.

## 4. Materials and Methods 

### 4.1. Adult Rat hNSC Cultures and Treatments

Ready-to-use primary adult rat hNSCs (Chemicon, Billerica, MA, USA) were grown in Eagle’s minimal essential medium/F12 medium added with B27 supplement, L-glutamine, fungizone, penicillin-streptomycin, and 20 ng/mL basic fibroblast growth factor (bFGF). The tissue culture plastic- or glass-ware for culturing hNSCs were coated with 5 μg/mL lamin and 10 μg/mL poly-L-ornithine. All tissue culture reagents were procured from Gibco/Invitrogen (Carlsbad, CA, USA). The hNSCs incubated in a 5% CO_2_ humidified incubator at 37 °C were subcultured every three-four days. To determine whether AQ stimulates the proliferation of hNSCs, cells were treated with AQ (1, 10, 100, 1000, and 10,000 nM) for 8, 24, and 48 h. All experiments were conducted three times in duplicate.

### 4.2. Small Interfering RNA (siRNA) Knockdown Experiments

Rat Nurr1 siRNA duplexes were purchased with Scrambled siRNA from Origene (Cat no. SR513154, Rockville, MD, USA). In this reagent, three different siRNA strands were combined to target disparate regions of the Nurr1 mRNA to improve the knockdown efficiency. The Nurr1-targeted siRNA sequences were provided by the manufacturer: SR513154A—rGrCrArGrUrUrArArGrArCrArArArUrGrUrArArGrGrCrAAA, SR513154B—rGrGrArArGrArUrUrGrCrArArArUrGrUrArUrGrArUrGrGGA, and SR513154C—rArGrArUrGrArUrArCrUrCrArArCrArUrArUrCrCrArGrCAG. A Lipofectamine^®^ 2000 siRNA Transfection kit (Life Technologies, Rockville, MD, USA) was used to transfection of adult rat hNSCs, after which the cells were utilized 24 h later for functional studies. Then, 10 μM of AQ was treated for 8 and 24 h after functional studies.

### 4.3. MTT Assay

MTT assay was used to determining cell proliferation to evaluate the proliferative effect of AQ, as Nurr1 agonist. MTT assay following the manufacturer’s protocol (Sigma-Aldrich). In brief, we seeded adult rat hNSCs at a density of 1 × 105 cells/mL in 96-well plates. After 24 h, the medium was replaced with medium containing AQ (1, 10, 100, 1000, and 10,000 nM) or vehicle and incubated for 24 h. Then, 10 μL of MTT solution was instilled to the each well containing 100 μL of medium, followed by further incubation for 4 h at 37 °C. At the endpoint of the period of incubation, the medium was removed if the cells were attached, and the converted dye was solubilized with dimethyl sulfoxide (DMSO). A microplate reader (Molecular devices LLC., CA, USA) was used to measure the absorbance of respective group at 540 nm. All experiments were conducted three times in triplicate.

### 4.4. Fluorescence-Activated Cell Sorting (FACS) Analysis

Cell cycle distribution was examined by FACS analysis. Cells were pre-incubated with 50 mM PD98059 (MEK inhibitor) for 1 h or 10 mM LY294002 (PI3K inhibitor) for 30 min, and then treated with AQ 10 μM. Then, 1 × 10^6^ cells were harvested and fixed with 3.7% paraformaldehyde (PFA). After fixation, a 50 µg/mL propidium iodide-staining solution containing RNase A (BD Biosciences, San Jose, CA, USA) was added. After 30 min, the cells were filtered with a nylon mesh filter and the Stained cells were sorted by flow cytometry (Calibur^TM^, BD Biosciences). All experiments were conducted three times in duplicate.

### 4.5. Western Blot

Cells were lysed in a 20 mM Tris–HCl (pH 7.4) buffer containing140 mM NaCl, 50 mM NaF, 1 mM EDTA, 1 mM Na_3_VO_4_, 10 μg/mL aprotinin, 1 mM phenylmethylsulfonyl fluoride and 1% (w/v) Nonidet P-40. For the detection of Nurr1 and E2F1, the nuclear and cytoplasmic fractions of the cells were isolated using the Nuclear/Cytoplasmic Extraction Kit following the manufacturer’s protocol. Cell fractions were separated by SDS–PAGE on 12% polyacrylamide gels and electrotransferred onto PVDF membranes (Bio-Rad, Hercules, CA, USA). The membranes were blocked with a Tris-buffered saline buffer containing 1% nonfat dry milk and 1% BSA for 1 h. Then, membranes were incubated with the primary antibodies against the proliferating cell nuclear antigen (PCNA; Cell Signaling, Danvers, MA, USA; 1:1000), minichromosome maintenance complex component 5 (MCM5; Cell Signaling; 1:1000), Nurr1 (Santa Cruz Biotechnology, Dallas, TX; 1:1000), E2F1, cyclin D_1_, cyclin A, CDK2, p27^KIP1^, p57^KIP2^ (Santa Cruz Biotechnology; 1:1000), β-actin (Santa Cruz Biotechnology; 1:1000) and lamin A (Cell Signaling; 1:1000), for overnight at 4 °C. Membranes were developed for 1 h with the peroxidase-conjugated anti-rabbit IgG (Santa Cruz Biotechnology). The blots visualized by the ChemicDoc XRS system (Bio-Rad) were quantified by the Quantity One imaging software (Bio-Rad). All experiments were conducted three times with triplicate.

### 4.6. Immunocytochemistry

Adult rat hNSCs were fixed in 4% PFA for 20 min at 20–25 °C and washed twice with phosphate-buffered saline (PBS). Cells were permeabilized with PBS containing 0.4% Triton X-100 for 20 min, followed by blocking with TBS containing 10% normal goat serum and 0.02% Tween 20 for 1 h at room temperature. Primary antibody incubations were conducted at 4 °C overnight in TBS including 0.02% Tween 20 and 3% BSA. Cells were after overnight incubation at 4 °C with the primary antibody against CDK2 (1:1000) and p27^KIP1^ (1:500), then washed with PBS. Cells were incubated with Alexa Fluor 488-conjugated goat anti-rabbit IgG (Invitrogen; 1:400) or Cy3-conjugated goat anti-rabbit IgG (Jackson Immunoresearch, West Grove, PA; 1:400) for 4 h at 20–25 °C. Immunofluorescence of cells was imaged by the LSM 700 Meta confocal microscope (Carl Zeiss, AG, Oberkochen, Germany) and analyzed using ZEN software (Carl Zeiss).

### 4.7. Animals and Administration

Eight-week-old adult male C57BL/6 mice were obtained from Koatech (Pyeongtaek, South Korea) and acclimated for one week before the experiment. All animals were housed following the National Institute of Health’s guidelines for the care and use of Laboratory Animals (NIH Publications No. 8023, revised 1978). Experimental procedures were approved and reviewed by the Institutional Animal Care and Use Committee at Kyung Hee University Hospital in Gangdong (Approval Number: KHNMC AP 2019-008). For Nurr1 activation, the mice were injected intraperitoneally with 20 mg/kg AQ for 14 days at 12 h intervals (Figure S2). The AQ dose and treatment duration used in this study were obtained from reports of AQ-induced pharmacological stimulation of Nurr1 in rodents [23,38]. AQ was diluted with 0.9% saline (vehicle) and prepared just before administration (six mice per group). Two weeks subsequent to the last AQ injection, the period for neuronal maturation [33], the mice were euthanized for subsequent analysis. Exclusion criteria were based on KHNMC’s criteria, and no animal died during the experiment.

### 4.8. Real-Time Quantitative Polymerase Chain Reaction (RT-qPCR)

The total RNA from mice whole brains was extracted using the RNeasy Mini Kit (Qiagen, Hilden, Germany), according to the manufacturer’s protocol. Superscript II reverse transcriptase (Life Technologies) was used to reverse transcription of RNA. RNA was reverse transcribed at 42 °C with random hexamer priming. Real-time RT-PCR was used to ascertain the mRNA levels of PCNA, Cdc25a, and MCM5 in hNSCs using primers specific for PCNA, Cdc25a, and MCM5 (The Mouse Cell Cycle RT 2 Profiler PCR Array, Qiagen), and β-actin (sense: 5′-ATG GGT CAG AAG GAC TCC TAC G-3′ and antisense: 5′-AGT GGT ACG ACC AGA GGC ATA C-3′). Reactions were processed in the StepOnePlus Real-Time PCR System (Applied Biosystems, Foster City, CA, USA).

### 4.9. Brain Tissue Preparation

The mice were anesthetized with avertin (0.5 g tribromoethanol with 1 mL amylene hydrate in 40 mL 3rd distilled water) and transcardially perfused with 0.05 M PBS. Ice-cooled 4% PFA in 0.1 M PB was used to fix. Next, the brains were isolated and post-fixed in 0.1 M phosphate buffer (PB) including 4% paraformaldehyde for 20 h at 4 °C. To cryoprotection, isolated brains were subsequently submerged with 30% sucrose in 0.05 M PBS solution for three days at 4 °C. The brains were embedded in Surgipath^®^ frozen section compound (Leica Biosystems, Wetzlar, Germany) and sectionalized into serial 30 μm-thick coronal sections by CM1850 cryostat (Leica Biosystems). Afterward, cryoprotectant (25% ethylene glycol, 25% glycerol, and 0.05 M PB) was used to store the tissue sections. Sectionalized tissues were stored at 4 °C pending further analysis.

### 4.10. Immunohistochemistry and Quantification

Free-floating brain sections were washed in PBS briefly and incubated overnight at 4 °C with either rabbit anti-p27^KIP1^ antibody (1:100) or rabbit anti-CDK2 antibody (1:200) in blocking solution. After being washed three times with PBS, the tissues were incubated with either Alexa Fluor 594 donkey anti-rabbit IgG (1:400) or Alexa Fluor 488 donkey anti-rabbit IgG (1:400) for 1 h at 20–25 °C. The tissue sections were mounted on ProbeOn™ Plus Microscope Slides (Thermo Fisher Scientific Inc.) and cover-slipped with Fluoroshield™ with DAPI (Sigma-Aldrich) to counterstain the nuclei. The entire tissue sections were imaged with a Zeiss LSM 700 Meta confocal microscope (Carl Zeiss). Then, the immuno-positive cells were quantified using ImageJ software (NIH, Bethesda, MD, USA).

### 4.11. Statistical Analysis

All data were presented as the means ± S.E.M of three different experiments. One-way analysis of variance followed by with the Holm–Sidak method using SigmaStat software (Ver. 3.10, Systat Software, Inc., Point Richmond, CA, USA) were applied in data analysis. A *p*-value <0.05 was concluded statistically significant. Blinding and randomization of data were not performed in the study.

## 5. Conclusions

The pharmacological stimulation of Nurr1 by AQ in adult hNSCs caused upregulation of positive cell cycle regulatory molecules (cyclin D_1_, cyclin A, and CDK2,) and downregulation of negative cell cycle regulatory molecules (p27^KIP1^ and p57^KIP2^). The expression of cell cycle progression markers, such as PCNA, MCM5, Cdc25a, and E2F1, also increased. In summary, the present study showed that the pharmacological stimulation of Nurr1 by AQ treatment of adult rodent hNSCs regulates cell cycle-related molecules to promote cell cycle progression.

## Supplementary Materials

Supplementary materials can be found at https://www.mdpi.com/1422-0067/21/1/4/s1, Figure S1: AQ promotes cell cycle progression in adult rat hNSCs. After the cells were treated with 1 μM AQ, (A) the G0/G1 phase ratio, (B) S phase ratio, and (C) G2/M phase ratio in the total cells were time-dependently analyzed by FACS for 48 h and represented compared with the vehicle-treated control., Figure S2: Administration of AQ in C57BL/6 mice. AQ was administered by intraperitoneal injection for 14 days at 12 h intervals., Figure S3: AQ stimulates the proliferation of adult rat hippocampal NSCs. The expression of mRNA levels was time-dependently measured by FACS for 24 h after treatment of 10 nM AQ in adult rat hippocampal NSCs (* *p* < 0.05, ** *p* < 0.01 compared with the vehicle-treated control).

## Abbreviations

AD	Alzheimer’s disease
AQ	amodiaquine
bFGF	basic fibroblast growth factor
CDK DAPI DG DMSO Erk1/2 FACS hNSCs MCM5 moDG NR4A Nurr1 PB PBS PCNA PD PFA Rb RT-qPCR SGZ siRNA	cyclin-dependent kinases 4′,6-diamidino-2-phenylindole dentate gyrus dimethyl sulfoxide extracellular signal-regulated protein kinases 1 and 2 fluorescence-activated cell sorting hippocampal neural stem cells minichromosome maintenance complex component 5 molecular layer area of DG nuclear receptor subfamily 4A Nuclear receptor related-1 phosphate buffer phosphate-buffered saline proliferating cell nuclear antigen Parkinson’s disease paraformaldehyde retinoblastoma Real-time quantitative polymerase chain reaction subgranular zone Small interfering RNA
