# Supplementary material for: Pharmacological Stimulation of Nurr1 Promotes Cell Cycle Progression in Adult Hippocampal Neural Stem Cells
Source: Int J Mol Sci. 2019 Dec 18;21(1):4. doi: 10.3390/ijms21010004 (PMC6982043; doi:10.3390/ijms21010004)
Supplement: Supplementary file 1 [file ijms-21-00004-s001.pdf]

Supplementary Materials

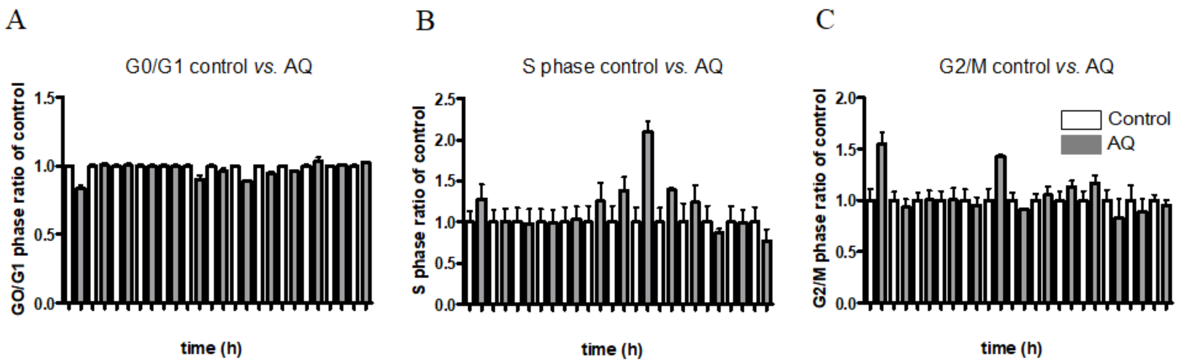

Figure S1. AQ promotes cell cycle progression in adult rat hNSCs. After the cells were treated with 1 μM AQ, (A) the G0/G1 phase ratio, (B) S phase ratio, and (C) G2/M phase ratio in the total cells were time-dependently analyzed by FACS for 48 h and represented compared with the vehicle-treated control.

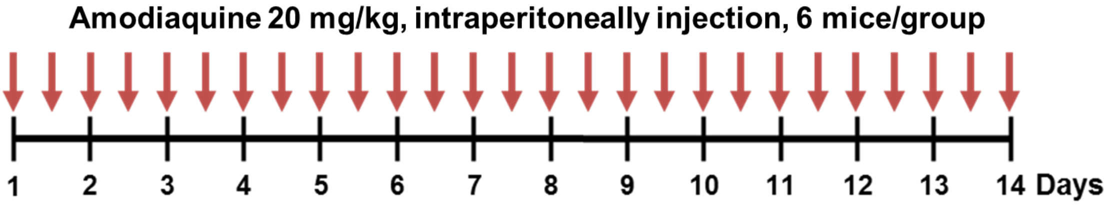

Figure S2. Administration of AQ in C57BL/6 mice. AQ was administered by intraperitoneal injection for 14 days at 12 h intervals.

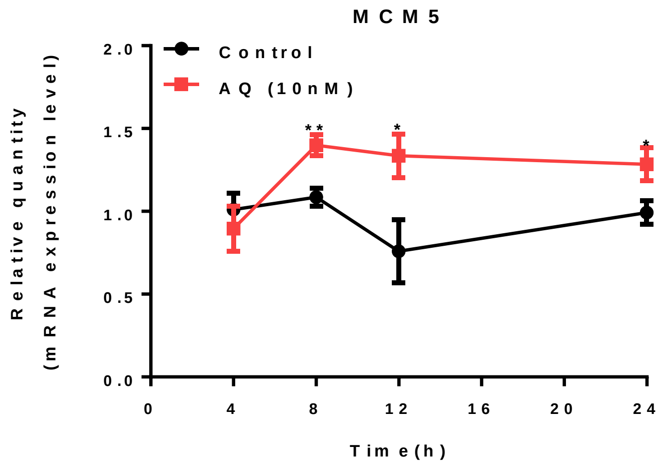

Figure S3. AQ stimulates the proliferation of adult rat hippocampal NSCs. The expression of mRNA levels was time-dependently measured by FACS for 24 h after treatment of 10 nM AQ in adult rat hippocampal NSCs ($^{*}p < 0.05$, $^{**}p < 0.01$ compared with the vehicle-treated control).
